# Biological Amnion Prevents Flexor Tendon Adhesion in Zone II: A Controlled, Multicentre Clinical Trial

**DOI:** 10.1155/2019/2354325

**Published:** 2019-04-03

**Authors:** Chunjie Liu, Jiangbo Bai, Kunlun Yu, Guoli Liu, Siyu Tian, Dehu Tian

**Affiliations:** ^1^Department of Hand Surgery, The Third Affiliated Hospital of Hebei Medical University, Shijiazhuang 050051, China; ^2^Department of Orthopedics, Tangshan Workers Hospital, Tangshan City 063000, China; ^3^Department of Orthopedics, The Second Hospital of Tangshan, Tangshan City 063000, China

## Abstract

**Introduction:**

Tendon adhesion to surrounding tissues is the most common complication reported after tendon repair. To date, effective solutions to prevent tendon injury are still lacking.

**Materials and Methods:**

A total of 89 patients with flexor tendon injury in zone II were recruited. The patients were divided into a control group, a poly-DL-lactic acid (PDLLA) group, and an amnion group according to the different tendon treatments applied. The control group was not subjected to other treatments. PDLLA and bioamniotic membranes were, respectively, used to wrap broken ends in the PDLLA and amnion membrane groups. The patients were followed at 1, 2, 3, 6, and 12 months after surgery and the ranges of active flexion and extension lag in the proximal and distal interphalangeal joints were evaluated.

**Results:**

The means of total active ranges of motion of the interphalangeal joints (excluding rupture cases) in the PDLLA and amnion groups did not significantly differ between each other but significantly differed from that of the control group. Statistical analysis showed a significant difference in the clinical grades of the outcomes among the control, PDLLA, and amnion groups. The incidence of complications in the control and PDLLA groups was found to be significantly higher than that in the amniotic membrane group; no significant difference was observed between the control and PDLLA groups.

**Conclusion:**

In this study, freeze-dried amniotic membrane transplantation was applied to promote healing of the flexor tendon in zone II and prevent adhesion. This technique presents a new method to solve the issue of tendon adhesion after repair.

**Clinical Trial Registration:**

The trial was registered by identifier ChiCTR1900021769.

## 1. Introduction

Advances in society and the industry have caused tendon injuries caused by trauma to become an extremely common phenomenon. Tendon adhesion to surrounding tissues is the most common complication reported after tendon repair [1]. According to statistics, over 320,000 cases of tendon injury are reported in the United States each year due to trauma and excessive exercise [2]. Tendon injury can considerably affect the quality of life of patients, and the number of reported injuries continues to increase. Clinicians have addressed this problem by implementing improved suturing techniques and early functional exercise. To date, effective solutions to prevent tendon injury are still lacking, and 30%–40% of all patients continue to experience complications, such as limited finger function. Adhesion after tendon injury repair is difficult to prevent in clinical practice [3]. Thus, reducing the incidence of adhesion after tendon repair without affecting tendon healing has become a popular research focus.

In-depth study of the mechanism of tendon adhesion formation has led to the development of a number of methods and materials to prevent tendon adhesion; the effects of improved suturing techniques, early rehabilitation training after operation, local application of drugs to inhibit inflammatory response, or inhibition of cytokine release, for example, have been explored. However, physical barriers to block exogenous healing are currently the main method implemented to prevent tendon adhesion [4–7]. Compared with nonabsorbable materials used previously, absorbable polymer compounds feature better biocompatibility and biodegradability. Tendon repair research has revealed that although these nonbiological material membranes can isolate tissues and prevent adhesion, they can also increase the possibility of tendon necrosis and permanent foreign body residue due to poor permeability and obstruction of nutrient penetration [8].

The amniotic membrane, a natural macromolecule material derived from organisms, is a semipermeable membrane that is smooth, nonvascular, nonnervous, nonlymphatic, and rich in matrix, cytokines, enzymes, and other active ingredients [9–11]. The unique structure of this membrane makes it an ideal biomaterial. Clinical and basic research on human amniotic membranes has a history of nearly 100 years. Demirkan et al. used fresh amniotic membrane to wrap the tendon of Leghorn chickens and achieved good results in preventing tendon adhesion [12]. However, fresh amniotic membranes cannot be preserved for a long time, as the corresponding preparation and quality control standards have not been established, and biosafety risks, such as hepatitis virus and HIV infection, continue to exist. As such, the clinical use of the amniotic membrane is limited.

Zone II consists of the region from the distal palmar crease to the insertion of the flexor digitorum superficialis. The zone's anatomy, with both tendons running within a fibro-osseous digital sheath, has been related to tendon adhesion following surgical repair. It used to be called “no man's land”. Surgeons have been trying to optimize postoperative outcomes. The study aimed to assess the clinical effectiveness of the amniotic membrane in preventing flexor tendon adhesion in zone II injuries by measuring the range of motion in treated fingers after tendon repair. This technique presents a new method to solve the issue of tendon adhesion after repair.

## 2. Patients and Methods 

### 2.1. Case Study

**Figure 1:**
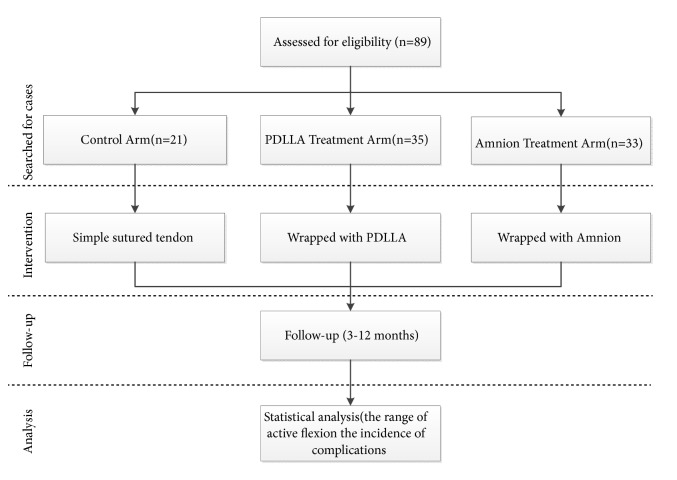
Study design diagrams.

A total of 89 patients with flexor tendon injury in zone II were recruited from the Third Affiliated Hospital of Hebei Medical University, Tangshan Workers' Hospital, and Tangshan Second Hospital from June 2015 to June 2018. The patients were divided into a control group, a poly-DL-lactic acid (PDLLA) group, and an amnion group according to the different tendon treatments applied (Figure 1). The patients included 50 males and 39 females with a total of 160 injured fingers and ages ranging from 21 years to 65 years (average age, 42.1 years). The causes of injuries included sharp cut wounds in 40 cases, electric saw injuries in 29 cases, and machine crush injuries in 20 cases. Injuries involved the index finger (44 fingers), middle finger (57 fingers), ring finger (45 fingers), or little finger (14 fingers). The time from injury to operation was 1–6 h (average, 4.5 h; Table 1).

All protocols were approved by the Ethics Board of the Third Hospital of Hebei Medical University, and patients and their families enrolled in the study were asked to sign an informed consent form. A total of 89 patients were recruited according to the criteria described in Table 2. Patients with repeated rupture of the repaired tendon were excluded from the analyses at all times of assessment.

### 2.2. Surgical Procedure

**Figure 2:**
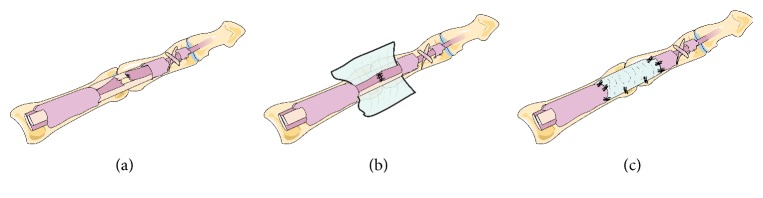
Operative technique for the application of the amniotic membrane allograft. (a) Exposure of the zone 2 FDP injury. (b) The tendon has been sutured, and the membrane is placed between the flexor tendons. (c) The membrane is wrapped around the FDP tendon and fixed to the remaining tendon sheath.

Local anaesthesia (45 cases) or brachial plexus block anaesthesia (44 cases) was administered. The wound was thoroughly debrided and extended by a Z-shaped incision on the palmar side. The flexion injury was extended to the distal end and extension injuries were extended to the proximal end to expose the tendon ends. The ends of the flexor tendon were restored, and some sheath tubes were opened for tendon repair. If the broken end of the tendon became fragmented and rough due to electric sawing or blunt extrusion, the tendon was repaired sharply. The length of tendon was shortened by no more than 1 cm. All operations were performed in the same manner, and all surgeons had similar levels of expertise and seniority. The states of the A2/A4 pulleys or tendon sheaths were noted during surgery and impaired as much as possible. All digital nerve injuries were repaired with the standard microsurgical technique, and the rehabilitation protocol was not altered. A 4-0 suture was used to repair tendons via the modified Kessler method. The wound was completely haemostatic, and the control group was not subjected to other treatments. PDLLA (Chengdu Dikang Zhongke Biomedical Material Co., Ltd.) and bioamniotic membranes (Jiangxi Ruiji Biotechnology Co., Ltd.) were respectively used to wrap broken ends in the PDLLA and amnion membrane groups. A 6-0 suture line was used to fix the biological amniotic membrane to the broken ends of the tendon. Postoperative plaster fixation of the wrist joint flexion at 30°, metacarpophalangeal joint flexion at 40°–50°, and interphalangeal joint flexion at 30° were carried out to limit wrist and finger extension (Figure 2).

### 2.3. Postoperative Treatment

All patients were instructed by hand therapists to follow the same rehabilitation programme and were asked to be available for evaluation. The rehabilitation was divided into three stages. In stage one (days 3–14), digital extension was prevented by a plaster, and a twice-daily passive motion program permitting nearly complete interdigital joint extension and flexion was carried out. In stage two (days 15–28), separate passive exercises for the proximal and distal interphalangeal joints were used in order to separate the profundus and superficialis repair sites. In stage three (day 29 onwards), active extension and flexion exercises were encouraged. The motion of the patient gradually returned to normal activities at 12 weeks. Use of the hand in simple activities of daily living was allowed only after 4 weeks. The follow-up period ranged from 3 months to 12 months.

### 2.4. Assessment

The patients were followed up 1, 2, 3, 6, and 12 months after surgery by senior surgeons of the hand department. The ranges of active flexion and extension lag in the proximal and distal interphalangeal joints were evaluated using the method described by Strickland and Glogovac (1980) with a digital goniometer [13]. Measurements were performed three times, and the average of these measurements was reported as the result. Total active motion (TAM) of the interphalangeal joints in comparison with that of the normal contralateral finger was calculated, and the results were classified according to the original Strickland grades (Table 3):

(1) Active PIP+DIP flexion−PIP+DIP losses of extension175°×100.

Complications, such as itching, erythema, exudate, and rupture, were noted.

### 2.5. Statistical Analysis

The data were analysed by SPSS 24 software, and the Kolmogorov–Smirnov test was used to check whether the data conformed to a normal distribution. The SNK test was performed to compare the mean active ranges of motion between the three groups. The proportion of fingers with excellent, good, fair, or poor function according to Strickland were analysed via the Kruskal–Wallis and Mann–Whitney tests. A participant with multiple occurrences of an adverse incident was counted only once. The incidence of adverse events was analysed with the chi-square test, and differences were considered significant if the p-value was less than 0.05. All tests were two-tailed.

## 3. Results

**Figure 3:**
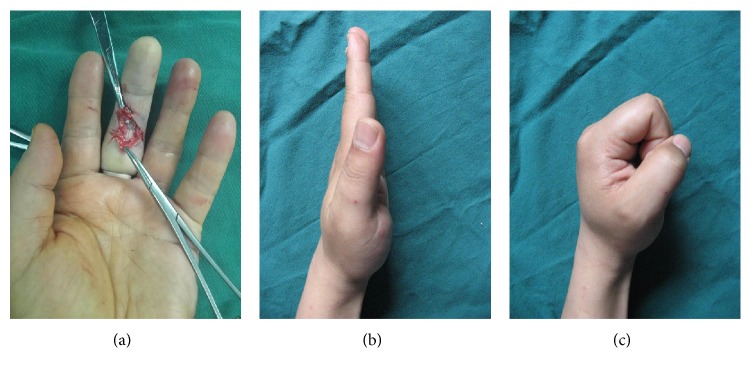
Case 1: ruptured tendon wrapped with a biological amniotic membrane. (a) Intraoperative view of the flexor tendons in zone 2. (b), (c) Follow-up visit: excellent aspect of the scar and functional recovery.

**Figure 4:**
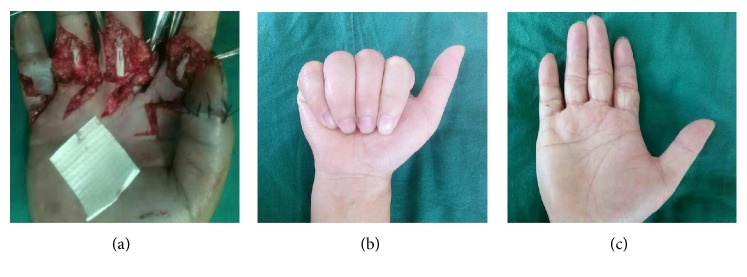
Case 2: ruptured tendon wrapped with a biological amniotic membrane. (a) Intraoperative view: the flexor tendons in zone 2. (b) and (c) Follow-up visit: excellent aspect of the scar and excellent functional recovery.

Between June 2015 and June 2018, a total of 89 patients with 160 injured fingers were recruited to the study. A total of 21, 35, and 33 patients were included in the control, PDLLA, and amnion groups, respectively. No significant differences between the three groups in terms of baseline characteristics were observed (Table 1). Two patients in the control and PDLLA groups were excluded from the ranges of active flexion because of rupture of the tendon repair. Postoperative follow-up revealed that 83.6% of the treated fingers in the amnion group exhibited excellent and good recovery according to the original Strickland grades, as shown in Figures 3 and 4, respectively.

### 3.1. Range of Active Digital Motion

**Figure 5:**
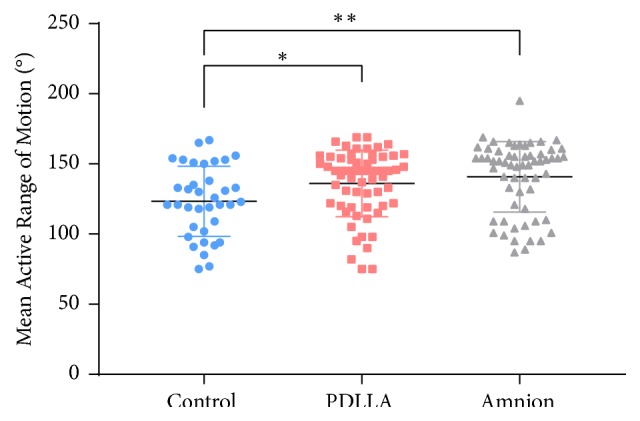
Mean range of motion at follow-up assessments by visit. The PDLLA group (n=34) and amnion group (n=33) were significantly different from the control group (n=20). *∗*: p<0.05; *∗∗*: p<0.01.

The means of total active ranges of motion of the interphalangeal joints (excluding rupture cases) in the control, PDLLA and amnion groups were 123.4°, 136.1°, and 140.8°, respectively. The results of the PDLLA and amnion groups did not significantly differ between each other but significantly differed from that of the control group (Figure 5).

**Figure 6:**
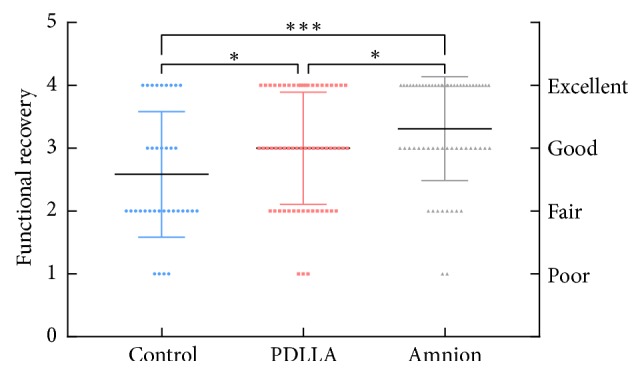
According to Strickland and Glogovac grading (1980), the functional recovery of flexor tendons in zone II (160 fingers). The amnion group (n=33) was significantly different from the control group (n=21) and PDLLA group (n=35). *∗*: p<0.05; *∗∗*: p<0.01, *∗∗∗*: p<0.001.

Outcomes according to the original Strickland grades are shown in Figure 6. The control, PDLLA, and amnion groups demonstrated 16, 43, and 51 excellent and good results, respectively. Statistical analysis showed a significant difference in the clinical grades of the outcomes among the control, PDLLA, and amnion groups.

### 3.2. Complication Assessment

Several complications, including repeated tendon rupture, itching, erythema, oedema, and exudate, may occur after tendon repair, especially after PDLLA or amnion implantation in vivo. In this study, the incidence of complications in the control and PDLLA groups was found to be significantly higher than that in the amniotic membrane group; no significant difference was observed between the control and PDLLA groups (Table 4).

### 3.3. Postoperative Rupture

A total of two cases of postoperative tendon rupture, including one case in the control group and one case in the PDLLA group, were found during follow-up. The ruptures occurred because of sudden and unexpected overload of the tendon. Reoperation determined that the mechanism of failure could be attributed to poor tendon healing. An evident aseptic inflammatory reaction was found at the healing site of tendon rupture in the PDLLA group.

## 4. Discussion

Among limb injuries, hand injury occurs relatively frequently; tendon injuries alone or in combination with other injuries account for approximately 30% of all hand injuries reported. The anatomical structure of the flexor tendon in zone II is complex, fine, and often associated with tendon sheath defects. This zone is a common site of adhesion formation after tendon injury repair.

Tendon adhesion is closely related to tendon healing. The healing process after tendon injury comprises two mechanisms: endogenous healing and exogenous healing. Endogenous healing is accomplished by stimulating the self-proliferation and migration of tendon cells on the surface and inside of the tendon through synovial fluid and cytokines. Exogenous healing, by comparison, is accomplished by fibroblast proliferation in the sheath and subcutaneous tissue around the tendon; these cells grow into the cross-section of the tendon along with capillaries of granulation tissue [14]. The strength of the tendon mainly depends on the endogenous repair mechanism. Adhesion during tendon healing can be attributed to several factors. Firstly, during exogenous healing, fibroblasts grow from the surrounding tissue to the broken end of the tendon, forming adhesion between the tendon and surrounding tissue; this process is the main mechanism of adhesion formation. Secondly, exudation of local tissue increases due to inflammatory reactions, and adhesion of the tendon is aggravated after mechanisation. Thus, the exogenous mechanism should be initiated earlier than the endogenous mechanism [15]. The exogenous mechanism occurs at the injury site during the inflammation stage and aggravates tendon adhesion after mechanisation. This phenomenon can easily lead to extensive adhesion formation exogenous tendon repair.

Blocking exogenous healing by using a physical barrier is currently the main method applied to prevent tendon adhesion. Many types of biological and nonbiological materials are used to repair the tendon sheath or as substitutes to prevent tendon adhesion. The mechanisms and clinical effects of these materials vary because of their different physical and chemical properties. However, comprehensive research has revealed that although nonbiological material membranes can isolate tissues and prevent adhesion, these materials also increase the possibility of tendon necrosis and permanent foreign body residue due to poor permeability and obstruction of nutrient penetration (S2 A). PDLLA is a kind of polylactic acid macromolecule material, which eventually degrades into carbon dioxide and water in vivo. It is nontoxic and harmless to human body. PDLLA is extensively utilised in clinical practice because of its good biocompatibility and biodegradability [16]; it achieves good clinical results and is one of the most widely used synthetic biodegradable polymers in medicine. PDLLA was included in the present study and compared with the amniotic membrane group with broad representation.

Pure PDLLA materials have good biocompatibility and biodegradability, but lack of active functional groups, poor permeability, and poor affinity to cells. The incidence of adverse events in the PDLLA group was considerably higher than that in amnion group. In the case of reoperation of the tendon rupture, the tendon was found to be poorly healed and accompanied by a severe aseptic inflammatory response. This finding is highly correlated with PDLLA implanted in vivo.

PDLLA and bioamniotic membranes prevent tendon adhesion mainly through physical isolation, but bioamniotic membranes are easier to permeate nutrients and release growth factors to promote tendon healing. In theory, bioamniotic membranes are more effective in preventing tendon adhesion. Similar results were obtained in the mean range of tendon motion between the two groups in this study, which may be related to the small sample size and the lack of early ultrasound or MRI data. Demirkan et al. used fresh amnions to wrap the tendon of chickens. Compared with the control group, histological observation showed that tendon fibers arranged in an orderly manner, with fewer adhesion tissues and better healing quality 3-6 weeks after operation. These results suggest that ultrasound and MRI should be performed regularly to evaluate the quality of tendon healing in clinical treatment. According to the results of the examination, patients are encouraged to take early activities. Early exercise can better reduce tendon adhesion.

The ideal materials for preventing tendon adhesion should feature complete absorption, good histocompatibility, and good permeability; it should not affect the quality of tendon healing whilst blocking the exogenous tendon healing and should contain factors that promote cell adhesion, growth, proliferation, and differentiation. The amniotic membrane, a natural macromolecule material derived from organisms, is a semipermeable membrane that is smooth, nonvascular, nonnervous, nonlymphatic, and rich in matrix, cytokines, enzymes, and other active ingredients. The unique structure of this membrane makes it an ideal biomaterial (S2 B).

The human amniotic membrane is a translucent bilayer membrane attached to the foetal surface of the placenta. This membrane evolves from the cytotrophoblast and is approximately 0.02–0.05 mm thick; thus, this membrane is the thickest basement membrane of the human body. The amniotic membrane has a smooth surface and a certain elasticity; it has no blood vessels, nerves, or lymph. The human amniotic membrane can be divided into the epithelial, basement membrane, compact, fibroblast, and sponge layers (S3). Under the electron microscopy, the epithelial cells of the amniotic membrane are connected by desmosomes, and there are many microvilli on the side of the cells. They form a complex labyrinthine duct system between the epithelial cells of the amniotic membrane. Semidesmosomes on the basement membrane are highly developed, and there are layers of fibers passing through the matrix. It contains a variety of growth factors and soluble molecules, collagen, laminin, fibronectin, proteoglycan, and other components that can promote cell adhesion and growth. Abundant collagen fibers reinforce tensile force; special structure of basement membrane surface makes epithelial cells easy to grow and adhere; labyrinth duct system on amniotic membrane surface makes it easy to exchange substances. The presence of these materials contributes to the extensive potential use of human amniotic membranes.

Clinical and basic research on the human amniotic membrane has a history of nearly 100 years. As early as in 1913, stem used the amniotic membrane for skin burn and ulcer wound transplantation. Subsequently, the amniotic membrane was used in ophthalmology, neurosurgery, and gynaecology as a dressing [17–19]. Studies have found that human amniotic membrane cells present the characteristics of embryonic stem cells and are also differentiated into three different types of embryonic cells; amniotic membrane cells show low immunogenicity and immunosuppressive effects and do not induce rejection or tumorigenicity after transplantation. These cells can produce many growth factors, such as transformed growth factor-*β*1 (TGF-*β*1), fibroblast growth factor (FGF), insulin-like growth factor-1, vascular endothelial growth factor, platelet-derived growth factor, and tissue inhibitor of metalloproteinase, all of which are important regulators for the repair of tendon injury and adhesion formation [20–22].

However, fresh amniotic membrane cannot be preserved for a long time, and potential biological safety hazards, such as hepatitis virus and HIV contamination, substantially limit its clinical use [23]. After dehydration and micronization of fresh amnion, Gellhorn prepared suspension before injection to treat tendonitis and arthritis and achieved good results in relieving pain and improving function [24]. In this study, fresh amniotic membrane was treated by lyophilisation, which completely preserves the amnion matrix structure and a variety of growth factors inherent in amniotic membrane. This technology addresses the technical bottleneck of amniotic membrane preservation and enables usage of the amnion as a natural biological substitute material.

Woodall implanted an amnion matrix graft with a tendon graft during anterior cruciate ligament (ACL) reconstruction and good clinical results have been achieved. This procedure used the proposed anti-inflammatory, scaffolding, and stem cell-producing effects of the amniotic membrane to biologically augment the healing process [25]. According to Strickland and Glogovac grading (1980), the functional recovery in the amniotic membrane group was considerably higher than those of the PDLLA and control groups. We believe that flexor tendon rupture can be isolated from the surrounding tissue after wrapping with the amniotic membrane to prevent or reduce tendon adhesion. The injured tissues were shaped in the form of scaffolds to improve the arrangement and shape of collagen fibers in the tendon cells. Nutrients penetrate through semipermeable membranes to promote the repair of injured cells and effectively enhance the endogenous healing of tendons. bFGF, HGF, and TGF-*β*1 promote the differentiation, transformation, proliferation, and migration of stem cells and inhibit the growth of TGF-*β* receptors, which can prohibit the proliferation and differentiation of fibroblasts; downregulate the expression of *α*-smooth muscle actin, fibronectin, and integrin; reduce the biological function of fibroblasts and decrease fibrosis and scar formation [26, 27].

In clinical applications, freeze-dried amniotic membranes demonstrate brittleness, insufficient mechanical strength, and rapid dissolution, causing slippage. Introducing other materials or changing the morphology of the amniotic membrane is therefore necessary to overcome the aforementioned limitations this material and increase its strength, extensibility, and moderate hydrophobicity to maximise the potential of amniotic tissue. Secondly, the outpatient follow-ups did not do ultrasound examination, which can further determine the quality of tendon healing, edema, and other complications. In addition, the sample size in present the study is fairly small; thus, increasing the sample size to verify our findings is necessary.

## 5. Conclusions

Clinical use and comparison with the control group revealed that the biological amniotic membrane presents good biocompatibility, excellent repair function, and no cytotoxicity or side effects on local tissues or the human body. The membrane inhibits exogenous invasion of the tissue and releases various cytokines to accelerate endogenous healing of the tendon. Taking the results together, the biological amniotic membrane may be considered a safe, effective, and completely absorbable barrier material that can effectively reduce tendon adhesion.

## Figures and Tables

**Table 1:** Summary of relevant demographic and clinical data.

Groups		Control	PDLLA	Amnion	Difference
		n=21	n=35	n=33	p-value
Mean age, years		42.2	40.9	43.2	0.675
Gender	Male	12	20	23	0.500
	Female	9	15	10	
Hand, n	Right	12	25	19	0.408
	Left	9	10	14	
Finger, n	2nd	11	15	18	0.991
	3rd	13	23	21	
	4th	9	18	18	
	5th	4	6	4	
Flexor tendon	Superficial and deep	26	47	49	0.743
	Deep	10	15	13	
Pulley, n	Unimpaired	35	49	43	0.619
	Impaired	7	13	7	

n=number of patients; PDLLA: poly-DL-lactic acid.

**Table 2:** List of inclusion and exclusion criteria of the study.

Inclusion criteria	Exclusion criteria
(i) Complete rupture of flexor tendon in zone II	(i) Thumb injured
(ii) Injury to surgery less than 6 hours	(ii) Patients with vascular injured requiring revascularization
(iii) The soft tissue allowed direct skin closure	(iii) Concomitant phalanx fractures or other injuries needing immobilization
(iv) Only one injured hand	(iv) Loss of skin substance requiring grafts or flaps
(v) Written informed consent to undergo the surgical procedure	(v) Uncompensated diabetes, neoplasia, haemocoagulative alterations, psychic disorders
(vi) Patients of either sex aged between 21 and 65 years	(vi) Smokers

**Table 3:** Strickland's original classification system^1^.

Group	PIP + DIP return (%)	(PIP+DIP flexion) – extension loss (degree)
Excellent	85-100	150+
Good	70-84	125-149
Fair	50-69	90-124
Poor	<50	<90

1 Strickland and Glogovac (1980).

**Table 4:** Incidence of complications. A participant with multiple occurrences of an adverse incident is counted only once. Significant if p < 0.05.

Groups	n	Itch	Erythema	Exudate	Rupture	P values
Control	21	0	3	2	1	P1=0.483
PDLLA	35	1	3	2	1	P2=0.030
Amnion	33	0	1	0	0	P3=0.007

n=number of patients; p1: control group and PDLLA group; p2: PDLLA group and amnion group; p3: amnion group and control group.

## Data Availability

The data used to support the findings of this study are included within the supplementary information file(s).

## Supplementary Materials

Supplementary Materials S1 Table: summary of relevant initial data. S2 Figure: difference between PDLLA membrane and bioamniotic membrane. (a) The PDLLA materials have good biocompatibility and biodegradability, but poor permeability and cell affinity. (b) The amniotic membrane allows the penetration of nutrients and releases a variety of growth factors. S3 Figure: division of the amniotic membrane into the epithelial, basement membrane, compact, fibroblast, and sponge layers.
