# Supplementary material for: Biological Amnion Prevents Flexor Tendon Adhesion in Zone II: A Controlled, Multicentre Clinical Trial
Source: Biomed Res Int. 2019 Apr 3;2019:2354325. doi: 10.1155/2019/2354325 (PMC6470416; doi:10.1155/2019/2354325)
Supplement: Supplementary Materials — S1 Table: summary of relevant initial data. S2 Figure: difference between PDLLA membrane and bioamniotic membrane. (a) The PDLLA materials have good biocompatibility and biodegradability, but poor permeability and cell affinity. (b) The amniotic membrane allows the penetration of nutrients and releases a variety of growth factors. S3 Figure: division of the amniotic membrane into the epithelial, basement membrane, compact, fibroblast, and sponge layers. [file 2354325.f1.zip › Supplementary Material S1.pdf]

Summary of relevant initial data

Number	Gender	Age	Cause of injury	Finger	Follow up (month)	Both sides	PIP(Proximal interphalangeal joint)°	DIP(Distal interphalangeal joint)°	TAM(Total active movement)°	Hospital	Group	Deep and superficial tendons	Complications
1	Male	32	Sharp injury	Index	3	Right	88	69	157	Third Hospital	PDLLA	Yes	
2	Male	25	Electric saw injury	Middle、Ring	6	Right	Middle 80 Ring 75	Middle70 Ring 75	Middle150 Ring 145	Third Hospital	PDLLA	Yes	
3	Female	39	Machine crush injury	2-5fingers	12	Right	Index70Middle73Ring78Little68	Index50Middle56Ring53Little52	dex120Middle129Ring131Little1	Third Hospital	PDLLA	Yes， Little：No	Itch
4	Female	60	Sharp injury	Middle	3	Right	85	60	145	Third Hospital	PDLLA	Yes	
5	Male	43	Electric saw injury	Middle、Ring	4.5	Right	Middle 81 Ring 82	Middle 69 Ring 73	Middle 150 Ring 155	Third Hospital	PDLLA	No	
6	Female	31	Sharp injury	Little	3	Right	90	79	169	Third Hospital	PDLLA	No	
7	Male	35	Sharp injury	Index、Middle	5	Left	Index87Middle90	Index75Middle76	Index162Middle166	Third Hospital	PDLLA	No	
8	Male	26	Electric saw injury	2-4fingers	6	Left	Index80Middle83Ring 75	Index68Middle71Ring 65	Index148Middle154Ring 140	Third Hospital	PDLLA	Yes	Middle：redness and swelling
9	Female	38	Electric saw injury	IndexMiddle	6	Left	Index82Middle87	Index79Middle76	Index161Middle163	Third Hospital	PDLLA	Yes	
10	Female	31	Sharp injury	Ring	3	Left	85	71	156	Third Hospital	PDLLA	Yes	
11	Male	48	Machine crush injury	Index-Middle	6	Right	Index72Middle74	Index65Middle68	Index137Middle142	Third Hospital	PDLLA	Yes	
12	Female	48	Machine crush injury	3-5fingers	12	Left	Index62Middle69Ring 58	Index57Middle50Ring 55	Index119Middle119Ring 113	Third Hospital	PDLLA	Yes	
13	Male	52	Electric saw injury	Middle	3	Right	88	76	164	Third Hospital	PDLLA	Yes	
14	Male	56	Sharp injury	Index	3	Right	85	71	156	Third Hospital	PDLLA	No	
15	Male	49	Sharp injury	Middle、Ring	6	Right	Middle75Ring 70	Middle70Ring 65	Middle145Ring 135	Third Hospital	PDLLA	No	
16	Male	43	Electric saw injury	IndexMiddle	6	Left	Index80Middle70	Index65Middle60	Index145Middle130	Third Hospital	PDLLA	No	
17	Female	41	Sharp injury	Little	3	Left	83	70	153	Third Hospital	PDLLA	Yes	
18	Female	39	Sharp injury	Ring	3	Left	Ring 80	Ring 65	Ring 145	Third Hospital	PDLLA	Yes	
19	Male	43	Electric saw injury	IndexRing	3	Left	Index80Ring 70	Index65Ring 60	Index145Ring 130	Workers Hospital	PDLLA	Yes	Indexredness and swelling
20	Male	36	Machine crush injury	2-4fingers	12	Right	Index70Middle65Ring 68	Index45Middle40Ring 43	Index115Middle105Ring 111	Workers Hospital	PDLLA	Yes	Ring：exudate
21	Male	55	Electric saw injury	2-5fingers	12	Right	Index45Middle40Ring 50Little55	Index37Middle35Ring 40Little40	Index82Middle75Ring 90Little95	Workers Hospital	PDLLA	Yes	Middle：exudate
22	Female	21	Sharp injury	Little	3	Right	86	75	161	Workers Hospital	PDLLA	Yes	
23	Female	29	Sharp injury	MiddleRing	5	Right	Middle78Ring 75	Middle70Ring 70	Middle148Ring 145	Workers Hospital	PDLLA	No	
24	Female	33	Machine crush injury	Middle	3	Right				Workers Hospital	PDLLA	Yes	Rupture of tendon
25	Male	46	Machine crush injury	IndexMiddle	5	Right	Index62Middle70	Index60Middle52	Index122Middle122	Workers Hospital	PDLLA	Yes	
26	Male	39	Sharp injury	Ring	3	Right	73	70	133	Workers Hospital	PDLLA	Yes	
27	Male	47	Sharp injury	MiddleRing	3	Right	Middle83Ring 85	Middle70Ring 72	Middle153Ring 157	Second hospital	PDLLA	Yes	
28	Female	35	Electric saw injury	2-4fingers	12	Right	Index48Middle45Ring 30	Index50Middle53Ring 45	Index98Middle98Ring75	Second hospital	PDLLA	Yes	Indexredness and swelling
29	Female	46	Machine crush injury	Ring	6	Right	Ring 56	Ring 50	Ring 116	Second hospital	PDLLA	Yes	
30	Female	58	Sharp injury	Index	3	Right	76	70	146	Second hospital	PDLLA	No	
31	Male	47	Sharp injury	Middle	3	Right	85	70	155	Second hospital	PDLLA	No	
32	Male	40	Sharp injury	MiddleRing	6	Right	Middle75Ring 70	Middle73Ring69	Middle148 Ring 139	Second hospital	PDLLA	Yes	
33	Female	32	Machine crush injury	MiddleRing	6	Left	Middle80Ring 78	Middle65Ring 63	Middle145Ring 141	Second hospital	PDLLA	Yes	
34	Male	48	Electric saw injury	IndexMiddle	6	Right	Index89Middle80	Index80Middle77	Index169Middle157	Second hospital	PDLLA	Yes	
35	Male	43	Machine crush injury	Ring	6	Right	Ring 80	Ring 71	Ring 151	Second hospital	PDLLA	Yes	

1	Male	29	Electric saw injury	Middle、Ring	6	Left	Middle 80 Ring 75	Middle60 Ring 55	Middle140 Ring 130	Third Hospital	amnion	Yes	
2	Female	39	Machine crush injury	2-4fingers	12	Left	Index50Middle63Ring48	Index50Middle65Ring53	Index100Middle109Ring101	Third Hospital	amnion	Yes	Index：redness and swelling
3	Male	65	Sharp injury	Middle	3	Left	85	70	155	Third Hospital	amnion	Yes	
4	Male	43	Electric saw injury	Index、Ring	6	Right	Index 82 Ring 86	Index 67 Ring 73	Middle 149 Ring 159	Third Hospital	amnion	Yes	
5	Male	51	Sharp injury	Index	3	Right	89	80	169	Third Hospital	amnion	Yes	
6	Male	35	Sharp injury	Index、Middle	5	Right	Index87Middle83	Index75Middle71	Index162Middle154	Third Hospital	amnion	Yes	
7	Male	58	Electric saw injury	2-4fingers	6	Right	Index80Middle83Ring 75	Index68Middle71Ring 65	Index148Middle154Ring 140	Third Hospital	amnion	Yes	
8	Female	28	Electric saw injury	Index	6	Right	Index82	Index70	Index152	Third Hospital	amnion	Yes	
9	Male	51	Sharp injury	IndexRing	3	Right	Index80Ring 79	Index73Ring 70	Index153Ring 149	Third Hospital	amnion	No	
10	Male	38	Machine crush injury	2-4fingers	6	Right	Index71Middle60Ring 55	Index54Middle46Ring 40	Index125Middle106Ring 95	Third Hospital	amnion	Yes	
11	Female	38	Machine crush injury	3-5fingers	12	Left	Index52Middle72Ring 48	Index47Middle53Ring 53	Index99Middle125Ring 101	Third Hospital	amnion	Yes	
12	Male	63	Electric saw injury	Middle、Ring	6	Right	Middle75Ring 85	Middle77Ring 70	Middle152Ring 155	Third Hospital	amnion	No	
13	Male	26	Sharp injury	Index	3	Left	85	75	160	Third Hospital	amnion	No	
14	Male	59	Machine crush injury	3-5fingers	12	Left	Middle75Ring 70Little71	Middle65Ring 63Little61	Middle140Ring 133Little132	Third Hospital	amnion	Yes	
15	Female	43	Electric saw injury	IndexMiddle	6	Left	Index80Middle83	Index75Middle71	Index155Middle154	Third Hospital	amnion	No	
16	Male	58	Sharp injury	Index	3	Right	86	80	166	Third Hospital	amnion	No	
17	Male	39	Sharp injury	Ring	3	Right	Ring 86	Ring 65	Ring 151	Third Hospital	amnion	No	
18	Male	43	Electric saw injury	IndexRing	3	Left	Index86Ring 82	Index63Ring 61	Index149Ring 143	Workers Hospital	amnion	Yes	
19	Male	36	Machine crush injury	2-4fingers	12	Left	Index50Middle55Ring 48	Index45Middle40Ring 41	Index95Middle95Ring 89	Workers Hospital	amnion	Yes	
20	Female	46	Electric saw injury	IndexMiddle	3	Right	Index77Middle82	Index80Middle81	Index157Middle163	Workers Hospital	amnion	No	
21	Female	48	Electric saw injury	MiddleRing	6	Left	Middle83Ring 79	Middle69Ring 73	Middle152Ring 152	Workers Hospital	amnion	Yes	
22	Female	31	Sharp injury	Little	3	Right	87	79	166	Workers Hospital	amnion	Yes	
23	Male	48	Sharp injury	Ring	3	Left	Ring 86	Ring 75	Ring 161	Workers Hospital	amnion	Yes	
24	Male	55	Sharp injury	Middle	3	Right	87	76	163	Workers Hospital	amnion	Yes	
25	Male	35	Machine crush injury	2-5fingers	12	Right	Index73Middle61Ring66Little40	Index52Middle64Ring59Little47	dex125Middle125Ring125Little[illegible]	Workers Hospital	amnion	Yes	

26	Male	45	Machine crush injury	2-4fingers	12	Right	Index75Middle79Ring 83	Index79Middle75Ring 73	Index154Middle154Ring 156	Second hospital	amnion	Yes	
27	Female	23	Sharp injury	Middle	3	Right	87	80	167	Second hospital	amnion	Yes	
28	Male	35	Electric saw injury	IndexMiddle	6	Right	Index81Middle83	Index80Middle81	Index161Middle164	Second hospital	amnion	Yes	
29	Female	37	Sharp injury	Ring	3	Right	85	80	165	Second hospital	amnion	Yes	
30	Male	49	Electric saw injury	IndexMiddle	6	Right	Index72Middle75	Index69Middle72	Index141Middle147	Second hospital	amnion	Yes	
31	Male	37	Sharp injury	Middle	3	Left	Middle83	Middle73	Middle156	Second hospital	amnion	No	
32	Male	49	Electric saw injury	Middle	6	Left	75	50	125	Second hospital	amnion	Yes	
33	Female	47	Sharp injury	Ring	3	Left	87	78	165	Second hospital	amnion	No	

1	Male	23	Electric saw injury	2-4fingers	12	Right	Index50Middle47 Ring 40	Index35Middle30 Ring 35	Index85Middle77 Ring 75	Third Hospital	control	Yes	
2	Female	39	Machine crush injury	IndexMiddle	12	Right	Index50Middle53	Index41Middle56	Index91Middle109	Third Hospital	control	Yes	Index：redness and swelling
3	Male	65	Sharp injury	Middle	3	Right				Third Hospital	control	Yes	Rupture of tendon
4	Male	43	Electric saw injury	Index、Ring	6	Right	Index 62 Ring 56	Index 59 Ring 63	Middle 121Ring 119	Third Hospital	control	Yes	
5	Male	61	Sharp injury	Little	3	Left	85	80	165	Third Hospital	control	Yes	
6	Male	35	Sharp injury	Index、Middle	5	Left	Index70Middle50	Index60Middle39	Index130Middle89	Third Hospital	control	Yes	Index：exudate
7	Male	45	Electric saw injury	2-4fingers	6	Left	Index70Middle63Ring 71	Index65Middle60Ring 60	Index135Middle123Ring 131	Third Hospital	control	Yes	
8	Female	22	Sharp injury	Index	6	Left	Index82	Index70	Index152	Third Hospital	control	No	
9	Female	51	Sharp injury	IndexRing	3	Right	Index53Ring 45	Index41Ring 47	Index94Ring 92	Third Hospital	control	No	
10	Female	55	Machine crush injury	2-4fingers	6	Left	Index67Middle75Ring 65	Index54Middle46Ring 40	Index121Middle121Ring 105	Third Hospital	control	No	Ring：edema
11	Female	29	Sharp injury	Little	3	Right	80	71	151	Workers Hospital	control	No	
12	Female	49	Electric saw injury	Ring Little	6	Left	Ring 80Little86	Ring70Little70	Ring150Little156	Workers Hospital	control	Yes	
13	Male	53	Machine crush injury	MiddleRing	12	Right	Middle71Ring 66	Middle50Ring 52	Middle121Ring 118	Workers Hospital	control	No	Ring exudate
14	Female	49	Sharp injury	Index	3	Right	83	70	153	Workers Hospital	control	Yes	
15	Male	50	Sharp injury	Middle	3	Left	87	80	167	Workers Hospital	control	Yes	
16	Male	43	Electric saw injury	IndexMiddle	6	Left	Index73Middle71	Index65Middle61	Index138Middle132	Second hospital	control	Yes	
17	Female	32	Electric saw injury	Middle	3	Right	83	71	154	Second hospital	control	Yes	
18	Male	39	Machine crush injury	MiddleRing	6	Right	Middle70Ring 72	Middle63Ring 61	Middle133Ring 133	Second hospital	control	No	
19	Male	41	Sharp injury	Little	3	Right	83	70	153	Second hospital	control	Yes	
20	Male	37	Electric saw injury	MiddleRing	6	Right	Middle58Ring 53	Middle40Ring 41	Middle98Ring 94	Second hospital	control	Yes	Middle：redness and swelling
21	Female	26	Sharp injury	IndexMiddle	6	Left	Index61Middle57	Index50Middle45	Index111Middle102	Second hospital	control	Yes	

A total of 89 patients with flexor tendon injury in zone II were recruited from the Third Affiliated Hospital of Hebei Medical University, Tangshan Workers' Hospital and Tangshan Second Hospital from June 2015 to June 2018.The patients were divided into a control group, a poly-DL-lactic acid (PDLLA) group and an amnion group according to the different tendon treatments applied.The patients included 50 males and 39 females with a total of 160 injured fingers and ages ranging from 21 years to 65 years (average age, 42.1 years). The causes of injuries included sharp cut wounds in 40 cases, electric saw injuries in 29 cases and machine crush injuries in 20 cases. Injuries involved the Index finger (44 fingers), Middle finger (57 fingers), Ring finger (45 fingers) or Little finger (14 fingers). The time from injury to operation was 1–6 h.The patients were followed at 1, 2, 3, 6 and 12 months after surgery by senior surgeons of the hand department. The ranges of active flexion as well as the extension lag in the proximal and distal interphalangeal joints using the method described by Strickland and Glogovac(1980) with a digital goniometer were evaluated.
