# Supplementary material for: Biological Amnion Prevents Flexor Tendon Adhesion in Zone II: A Controlled, Multicentre Clinical Trial
Source: Biomed Res Int. 2019 Apr 3;2019:2354325. doi: 10.1155/2019/2354325 (PMC6470416; doi:10.1155/2019/2354325)
Supplement: Supplementary Materials — S1 Table: summary of relevant initial data. S2 Figure: difference between PDLLA membrane and bioamniotic membrane. (a) The PDLLA materials have good biocompatibility and biodegradability, but poor permeability and cell affinity. (b) The amniotic membrane allows the penetration of nutrients and releases a variety of growth factors. S3 Figure: division of the amniotic membrane into the epithelial, basement membrane, compact, fibroblast, and sponge layers. [file 2354325.f1.zip › Supplementary Material S2 S3.docx]

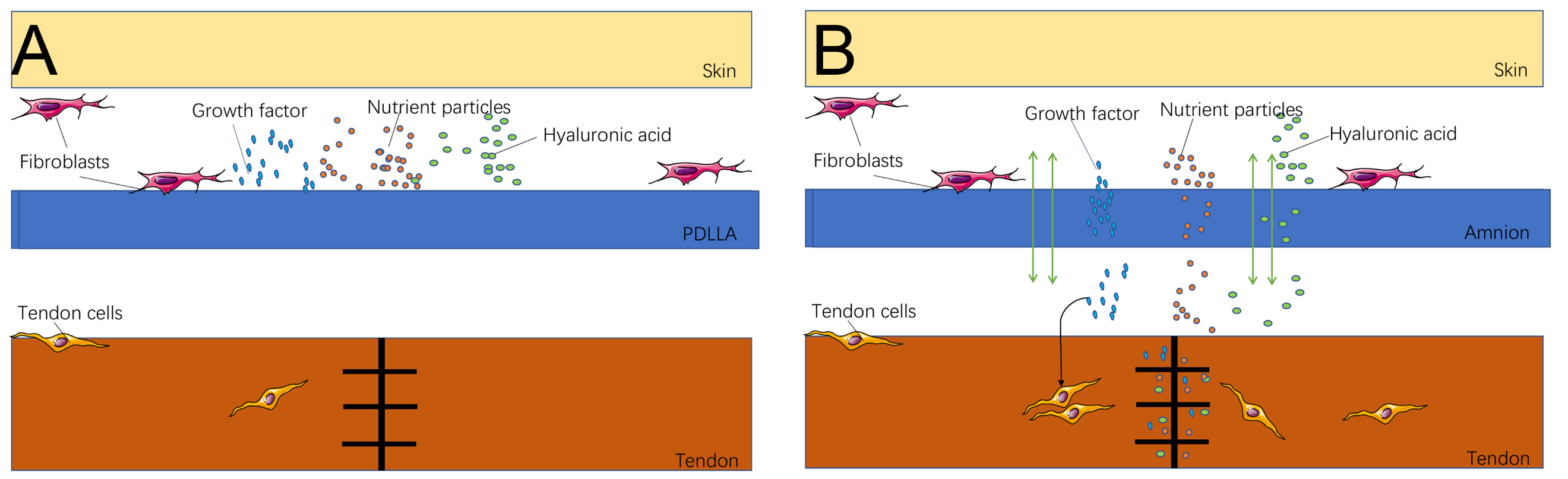


S2: Difference between PDLLA membrane and bioamniotic membrane. (a) The PDLLA materials have good biocompatibility and biodegradability, but poor permeability and cell affinity. (b) The amniotic membrane allows the penetration of nutrients and releases a variety of growth factors.


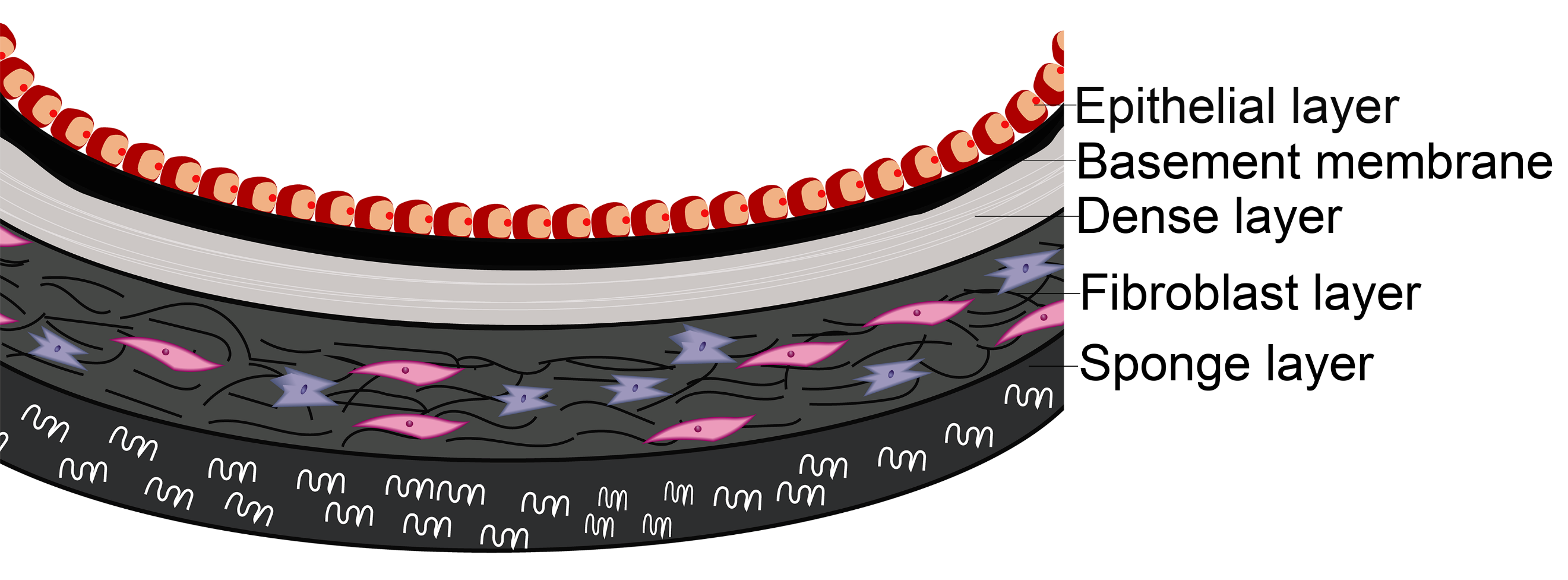


S3: Division of the amniotic membrane into the epithelial, basement membrane, compact, fibroblast and sponge layers.
